# Letter from Berlin

**Published:** 1904-05

**Authors:** Roswell Park

**Affiliations:** Berlin


### CORRESPONDENCE.

### Letter from Berlin.

DR. ROSWELL PARK GIVES SOME IMPRESSIONS OF THE RECENT CONGRESS OF GERMAN SURGEONS.

*Editor Buffalo Medical Journal*:

Sir—It has occurred to me that a brief account of the meeting of the German "Gesellschaft für Chirurgie," or Surgical Association, whose sessions, extending over four days, have just been completed, might be of some passing interest to your readers. This is the thirty-third annual meeting, therefore it will be seen that it is an older institution than our American Surgical Association. It is, however, in one respect, less select, since its membership is unlimited, and it is open to all those who confine themselves to surgical work; whereas the American society is limited, at present, to one hundred and twenty-five, it being the intent that only those who have already made their mark in surgery shall be considered as candidates. There are advantages in each way of doing things. For my own part I have always thought that a judiciously selected but unlimited membership list brings the greatest good to the greatest number. In this matter, however, I have so far been in the minority, though I have urged the "open door" policy.

The founders of the German society were Langenbeck, Billroth, Volkmann, Bruns, Bardeleben, Simon, Gurlt, Thiersch and Esmarch, and a picture of these men, whom we all delight to honor, hangs in the surgical clinic of the General Hospital. I have had the honor of being one of the four or five regular American members for a numbei of years, but have attended very few meetings. These are held during the week following Easter, which is vacation time in all the German universities, so that a large gathering is always possible. There are some four hundred and fifty members, and the program for the meeting included about one hundred and fifty papers. Thus it will appear to be an exceedingly active body of men. All of the surgeons of whom we ever hear belong to it, as well as many others.

Meetings are always held in Berlin, since here the society owns its own home. It stands on the grounds of the University Surgical Clinic; has an auditorium that will seat nearly five hundred, a very valuable library, numerous portraits, a museum, and all that can ever be needed for its purposes. The building, not including the land, cost one hundred and twenty-five thousand dollars, of which amount the grandmother of the present Kaiser contributed a very generous amount. Here meet numerous local and more national medical societies; the Langenbeck House, as it is called, is a sort of national surgeons' headquarters, somewhat corresponding to the Royal College of Surgeons in London; only the society has nothing to do with licensing or governing the practice of surgery.

Here, too, met during the past week the German Association of Orthopedic Surgeons, the meeting lasting only one day, under the presidency, this year, of Dr. Heusner, of Barmen. They had a program replete with interest, the principal subject for discussion being contractures of joints, while congenital dislocations, especially of the hip, were treated of by a number of writers. Lorenz, of pseudo-American fame, was not present. I was not a constant attendant, but I could not gather that *his* method of reducing congenitally displaced hips has won any great esteem over here. To be sure, however, the newspapers here are not generally on his side; neither is he taken so very seriously—at least not favorably—by the men here with whom I have talked.

To this orthopedic association belong about a hundred members. I note that among these the principal *operating* orthopedists belong to the surgical association, while those who work mainly or entirely with massage, apparatus and gymnastics, form a sort of group by themselves, as in the American Orthopedic Association. It is curious how men seem amiably separated according as their tastes and acquirements lead them to rapid operative or slow nonoperative methods. The operative method of treating lordosis or angular curvature was, however, not mentioned; were it still received with favor I think it would have been otherwise.

Inasmuch as the capacity of the auditorium is limited practically to the membership, admission to the surgical association was given only to members and invited guests; and then it was overcrowded, even at the opening session. The president, this year, was Prof. H. Braun. The first papers were by Körte, Franz, Riese, Lexer and Hoffmann, and concerned the surgery of the arteries and their surgical anatomy, especially in the osseous system. The most interesting portion of the morning was the demonstration by Hoffa, who is well known to us as an orthopedic surgeon, and by his book on fractures and dislocations, of some twenty cases, selected out of a large number, in which he had practised tendon plastic or transplantation methods. His demonstration was most interesting and most convincing as to the value of the procedure in many instances of paralytic deformity and loss of function.

The features of the first afternoon were papers on the method of preventing collapse of the lung during operations within the thorax. Sauerbruch, of Breslau, exhibited a pneumatic cabinet in which may be enclosed the head of a patient and the anesthetiser, while the air pressure is raised a little; or within which conditions may be reversed, so that the head of the patient is outside, while the operator and his assistants operate on the thorax from within the cabinet under a slightly reduced barometric or manometric pressure. The former is a temporary matter and submits the anesthetiser to a condition like that under which men work in caissons. The latter is quite new and experimental.

Mikulicz, in whose clinic this cabinet has been once tested, reported the loss of his patient. A much simpler method of establishing similar conditions was described by Petersen and Brauer, of Heidelberg, who also reported the loss of the only patient operated by their procedure. Both methods were demonstrated after the meeting, upon dogs, and both beautifully illustrated what was claimed for them,—*on animals.* Whether either of them is practical or practicable remains to be shown.

The balance of this afternoon session was devoted to the usefulness of the *x*-rays, in malignant disease, and to the nature of this disease. Petersen read a paper on the inoculation and implantation of cancer, but made it no clearer than before that the surgeons have been much more ready to accept the parasitic theory than the pathologists who work only in the dead-house and at the desk, i. e., that cancer is an infectious disease. Other opinions to the same effect were distinctly voiced by various speakers in public and private.

What can be done in the treatment of these cases by Röntgen rays and by radium was then illustrated by Lassar, the eminent dermatologist, who exhibited a remarkable collection of some forty or fifty patients thus treated. They all were cases of superficial lesions, some original, some recurrent. Of some of them he had microscopic sections, while of all he had beautifully colored wax models, showing the condition before commencing treatment. The results were beautifully demonstrative of the benefits obtainable in certain cases, especially the inoperable. And yet Lassar is himself unwilling to speak of them without great reserve —which was quite noteworthy. I do not think such a demonstration could be made anywhere else in the world; yet in spite of this and in the face of all these facts, there was a caution in his speech which should find many imitators. With us the temptation is too strong to regard two or three apparent successes as a final demonstration that *x*-rays will *cure* cancer. Nor was any attempt made to show that these lesions are not best treated by a clean excision, especially when seen reasonably early. Lassar, moreover, took pains to set forth numerous instances in which little or no benefit accrued—for example, in epithelioma of the tongue, where the lingual lesion might perhaps improve, but the deep infiltration surely would progress. Nevertheless, he showed a number of cases of recurring cancer of the breast, especially the disseminated and ulcerating forms, which had been greatly improved. Of radium he had but little to say, though that little was rather favorable.

The first evening was devoted to demonstrations with the projecting lantern. Krause, Lexer, and Hoffa all showed lantern slides, those of Lexer on the distribution of the bloodvessels in the bones being especially beautiful and exciting the greatest admiration. He injects the arteries with a mercurial preparation and then subjects the specimens to the Röntgen rays.

The second morning was practically devoted to the surgery of the upper abdomen. Kehr, who boasts that he has built up his little town of Halberstadt, reported on five new operations which he had practised on the biliary passages; i. e., resection of both ducts and a hepatico-duodenostomy, hepato-cholangio-enterostomy, ligature of the hepatic artery for aneurism, gastro-duodenostomy after closure of the upper part of the duodenum, and implantation of a pancreatic fistula in the gallbladder with a subsequent gastrocystotomy. I find that by some of the German surgeons Kehr is not taken very seriously, while by others, he is—very seriously. Körte, of Berlin, had a most interesting paper on the relations of biliary and pancreatic diseases; Ehrhardt talked entertainingly about the peritoneal infections, which arise from the biliary passages, and the venerable Bardenheuer, of Cologne, spoke on pancreatic hemorrhages. He, by the way, some years ago wrote a large book on the traction or extension treatment of fractures, which, as everyone at home knows, is absolutely of American origin.

The balance of the day was devoted to a miscellaneous program, which included many subjects, frequently illustrated with patients, and always with specimens or drawings.

The annual dinner was held at the Savoy at 5.30 p. m. In this respect our German friends set a good example. Only four speeches were made, and the company had separated by half-past eight. The advantages of such an arrangement are obvious.

The third day's session opened with a series of papers on bone surgery by a dozen different writers. This was followed by a sort of symposium on surgical diseases of the kidney, in which Kümmel and Krönlein dealt especially with tubercular lesions, and showed some remarkable specimens. Calculous disease received considerable attention. Ahrens reported a nephrotomy of a solitary kidney, while Barth showed a patient with horseshoe kidney upon which he had done an exploratory incision. Several writers then dealt with decapsulation of the kidney for Bright's disease—a method evidently not popular over here, for, so far as I gathered, no one mentioned it save to condemn it.

The third afternoon session was devoted to the peritoneum, stomach and intestinal canal. Mikulicz discussed the possibility of increasing the resisting power of the peritoneum; Payr reported on mesenteric thrombosis following intraäbdominal operations, and numerous papers were read on ulcer of the stomach, cancer of the pylorus and intestines, and the operative measures to be instituted for their relief. The writers were of one accord in at least this respect,—that all ulcerative lesions of the gastro-intestinal tract were best treated by operation of some kind.

The fourth morning session began with the apparently inevitable subject of appendicitis, which was discussed especially by Lauenstein, Federman, Cordua, Karewski and Sonnenburg. These are the men in all Germany who are, perhaps, most competent in this direction. Sonnenburg told me that he had himself operated on nearly eighteen hundred cases, which puts him on a plane with our best American surgeons, and entitles him to respectful hearing. He certainly has had larger experience with this condition than that of scores of his colleagues combined. During the session Brentano showed the specimen of a remarkable case of Hirschsprung's disease; i. e., congenital reduction in length and enlargement of caliber of the intestinal canal. It came from a boy of 14, who suffered from coprostasis to such extent that at one single time twenty-seven pounds of fecal matter were taken away from him. During the final afternoon session a variety of papers were read, one of them on so-called chorion-epithelioma of the testicle, by Holländer, while specimens and drawings were as numerous as ever.

Prof. Krönlein, of Zürich, was elected president for the ensuing year. He will be a great improvement upon Dr. Braun, of Göttingen, the retiring officer, who is old, feeble, toothless and hard to hear or understand, and who seemed to have little power of preserving good order during the meetings.

Altogether the meeting has been to me one of great entertainment. There has been little new, and nothing of tremendous interest, but there has been evinced a notable degree of research work and those rare, germanic traits of industry and never-tiring zeal. I have been making comparisons with the similar meetings of our own American Surgical Association, and do not feel that we need consider ourselves one whit inferior in any respect. Our meetings are more dignified, the papers are equally scientific, and our members much less prone to indulge in personalities or slighting and even insulting behavior. My regard for German surgery was comprehensive enough before I attended this meeting. I cannot say that it has been particularly enhanced thereby.

The American surgeons certainly lead the world today, and can teach their foreign colleagues many things. Some of the most progressive and least prejudiced of the continental surgeons are beginning to realise and acknowledge this, and a number of them are coming over to our World's Fair. Let us welcome them and impress them so far as we can, but let us not repeat the Lorenz fiasco. After all it repays our students to come to the continent, for they may learn what to avoid as well as what to do. It seems to me that the greatest advantage attendant upon studying surgery in the German clinics is the frequency with which one may follow cases from the operating table to the autopsy room, where their perplexities are so often cleared away.

The Anglo-American Medical Society meets every Saturday night in the Central Hotel, when I had the pleasure of addressing some fifty of my fellow countrymen. Every American coming here to study should ally himself at once with this excellent organisation, which can be of the greatest service to him. It is the only labor union I know of which serves to bring out the best work of the men who belong to it as well as of the various teachers in Berlin, who are anxious to do all they can to win the commendation and patronage of its members.

Roswell Park.

Berlin, April 10, 1904.

